# Genome-wide identification and analysis of the *IQM* gene family in soybean

**DOI:** 10.3389/fpls.2022.1093589

**Published:** 2023-01-06

**Authors:** Tianxiao Lv, Qiongrui Liu, Hong Xiao, Tian Fan, Yuping Zhou, Jinxing Wang, Chang-en Tian

**Affiliations:** ^1^ Guangdong Provincial Key Laboratory of Plant Adaptation and Molecular Design, Guangzhou Key Laboratory of Crop Gene Editing, Innovative Center of Molecular Genetics and Evolution, School of Life Sciences, Guangzhou University, Guangzhou Higher Education Mega Center, Guangzhou, China; ^2^ Suihua Branch Institute, Heilongjiang Academy of Agricultural Sciences, Suihua, Heilongjiang, China

**Keywords:** IQM, identification, family analysis, soybean, CaMBP

## Abstract

IQM, a plant-specific calmodulin-binding protein, plays multiple roles in plant growth and development. Although a comprehensive analysis has been carried out on the *IQM* family genes in *Arabidopsis* and rice, the number and functions of *IQM* genes in other species have not been explored. In this study, we identified 15 members of the soybean (*Glycine max*) *IQM* gene family using BLASTP tools. These members were distributed on 12 soybean chromosomes and constitute six pairs caused by fragment duplication events. According to phylogeny, the 15 genes were divided into three subfamilies (I, II, and III), and members of the same subfamily had similar gene and protein structures. Yeast two-hybrid experiments revealed that the IQ motif is critical for the binding of GmIQM proteins to GmCaM, and its function is conserved in soybean, *Arabidopsis*, and rice. Based on real-time PCR, the soybean *IQM* genes were strongly induced by PEG and NaCl, suggesting their important biological functions in abiotic stress responses. Overall, this genome-wide analysis of the soybean *IQM* gene family lays a solid theoretical foundation for further research on the functions of *GmIQM* genes and could serve as a reference for the improvement and breeding of soybean stress resistance traits.

## Introduction

Many signaling pathways and complex networks are involved in the response of plant to biotic and abiotic stresses (Saijo and Loo, 2020; Zhang et al., 2022). Ca^2+^ is an important second messenger that plays an important role in plant adaptation to external stimuli, thereby regulating multiple physiological processes and signaling transduction through Ca^2+^ sensors and their target proteins (Reddy et al., 2011; Carafoli and Krebs, 2016; Edel et al., 2017). The calcium sensors in higher plants include calmodulin (CaM), CaM-like proteins (CML), calcium-dependent protein kinase (CDPK), and calcineurin B-like proteins (CBL). Among these calcium sensors, CaMs are highly conserved acidic heat-stable proteins. There are seven CaMs in the model plant, *Arabidopsis* (*Arabidopsis thaliana*), and their amino acid sequences are highly similar, with only one to five amino acid differences (Williams, 1992; Chin and Means, 2000; DeFalco et al., 2009; Ranty et al., 2016; Aldon et al., 2018). In most cases, a single CaM has no biochemical or enzymatic activity, but acts by binding to Ca^2+^ and various downstream target proteins called CaM-binding proteins (CaMBPs), including kinases, cytoskeletal proteins, transcription factors, and metabolic enzymes that are involved in plant development, metabolic regulation, defense, and stress responses (Yang and Poovaiah, 2003; Bouché et al, 2005; Truman et al., 2013; Ali et al., 2020).

IQM has been identified as a calcium-independent CaMBP family with an IQ motif in *Arabidopsis* (Zhou et al., 2010). The IQ motif is the first recognized calcium-independent CaM-binding domain, and its complete amino acid sequence is IQxxxRGxxxR (Rhoads and Friedberg, 1997). In addition to the IQM family, there are four other classes of the IQ motif-containing protein family in plants: the myosin protein family (Reddy and Day, 2001), the calmodulin-binding transcription activator (CAMTA) family (Bouché et al., 2002), the cyclic nucleotide-gated channel (CNGC) family (Talke et al., 2003), and the IQ67-domain containing protein (IQD) family (Abel et al, 2005). Members of these five families differ in the number and distribution of IQ motifs. Compared to other families, only few studies have been performed on the function of the IQM family.

To date, the biological functions of *IQM* genes have only been investigated in *Arabidopsis*. There are six *IQM* members, *IQM1* to *IQM6*, in *Arabidopsis*. The N-terminal of the typical IQMs has a sequence homologous to the pea heavy-metal induced protein 6A (PHMIP 6A), in which the IQ motif is located, while their C-terminal has a fragment homologous to the ribosome inactivating protein, trichosanthin (Zhou et al., 2010). IQM1 plays an important role in the modulation of stomatal movement by affecting the ROS content (Zhou et al., 2012) and is a key regulator in plant disease defense mediated by JA signaling (Lv et al., 2019). AtIQM5 regulates flowering by modulating juvenile-to-adult transition (Gong et al., 2016) and promotes lateral root and callus formation by interacting with IAAs (Zhang et al., 2022). IQM4 affects seed dormancy and germination by regulating ABA biosynthesis and signaling in seeds (Zhou et al., 2018). Although the partial roles and internal mechanisms of IQMs in *Arabidopsis* have been revealed in these studies, research on the role of *IQM* family members in other species is still lacking. Besides *Arabidopsis*, whole genome analysis of the *IQM* family genes has only been performed for rice (Fan et al., 2021).

In this study, 15 non-redundant soybean (*Glycine max*) *IQM* genes were identified using bioinformatics analysis on a genome-wide scale and molecular biology techniques. The phylogenetic relationships, gene structure, conserved motifs, chromosome location, evolutionary pattern analysis, yeast two-hybrid analysis, and expression profile in response to abiotic stress and hormones of the soybean *IQM* family were discussed. Our results provide a theoretical basis for subsequent functional analysis of soybean *IQM* genes and insights for improving soybean resistance to biotic and abiotic stresses.

## Materials and methods

### Identification of soybean IQM proteins

Six *Arabidopsis* IQM protein sequences were used as reference, and their homologous sequences in soybean were retrieved from Phytozome v13 database (http://phytozome-next.jgi.doe.gov ) through Protein Basic Local Alignment Search Tool (BLASTP) program. The Pfamscan (https://www.ebi.ac.uk/Tools/pfa/pfamscan/ ) and SMART (http://smart.embl-heidelberg.de/ ) databases were used to annotate the conserved domains of candidate sequences. Finally, proteins containing the complete IQ motif were identified as members of the soybean IQM family. Information on soybean *IQM* genes, including CDSs, genome sequences, location coordinates, lengths of open reading frames (ORF), number of amino acids, and molecular weight, were acquired from Phytozome v13. The physicochemical characteristics of the GmIQMs were generated using ExPASy (http://web.expasy.org/protparam/ ). Subcellular localization was predicted using the WoLF PSORT program (http://wolfpsort.org ).

### Phylogenetic analysis

To examine the intraspecific and interspecific evolutionary relationships of *IQM* genes, the predicted IQM protein sequences of multiple species were obtained from the corresponding databases. *Arabidopsis* and rice IQM protein sequences were downloaded from TAIR (http://www.arabidopsis.org) and RAP-DB (http://rapdb.dna.affrc.go.jp/ ), respectively, whereas soybean, alfalfa, tomato, maize, sorghum, and *Brachypodium distachyon* IQM protein sequences were retrieved from *Glycine max Wm82.a2.v1, Medicago truncatula Mt4.0v1, Solanum lycopersicum ITAG4.0, Zea mays B84 v1.2*, *Sorghum bicolor v3.1.1*, and *Brachypodium distachyon v3.1* of Phytozome v13. ClustalW software was used for multisequence alignment. The parameters were set to the system default values [Pairwise Alignment—Gap Opening Penalty:10.00; Gap Extension Penalty: 0.10. Multiple Alignment—Gap Opening Penalty:10.00; Gap Extension Penalty: 0.20. Use Negative Matrix: off; Delay Divergent Cutoff(30)]. MEGA11 software was used to construct an unrooted phylogenetic tree using the neighbor-joining (NJ) method, and bootstrap analysis was conducted using 1,000 replicates (Wu et al., 2016). The generated phylogenetic tree was identified using iTOL (https://itol.embl.de/ ).

### Analysis of the structure of soybean *IQM* gene and protein

To compare the gene structures of *GmIQMs*, the distribution of exons and introns was analyzed using GSDS2.0 (http://gsds.cbi.pku.edu.cn) (Mei et al., 2021). The gff3 format files of soybean IQM genes downloaded from Phytozome v13 database were imported, then the system will automatically generate the structure chart contained CDS, UTR and Intro. To understand the similarities and differences in protein structures, the conserved motifs of encoded GmIQM proteins were identified using the MEME database (https://meme-suite.org/meme/ ), the maximum number of motifs was set to 15.

### Chromosomal localization and gene duplication

The distribution image of *GmIQM* genes on the soybean chromosome was generated by MG2C (http://mg2c.iask.in/mg2c_v2.1/ ) according to the gene information retrieved from the Phytozome v13 database. Gene duplication analysis was performed as previously described (Feng et al., 2014). The SoyBase browser (http://soybase.org/gb2/gbrowse/gmax1.01) was used to search for duplicate gene pairs. Coparalogs were considered to be duplicated in tandem if they were on the same chromosome and separated by five or fewer genes in a 100-kb region (Wang et al., 2010); otherwise, they were deemed to be fragment duplications. To further analyze the divergence of duplicated genes, the synonymous substitution rate (Ks) and non-synonymous substitution rate (Ka) were calculated using TBtools software. According to a rate of 6.1×10^-9^ substitutions per site per year, the divergence time (T) was calculated using the Ks value and the formula: T = Ks/(2×6.1×10^-9^)10^-6^ Mya (Lynch and Conery, 2000).

### Plant materials and growth conditions

The *Arabidopsis* culture conditions were obtained from Lv et al, 2019. *Arabidopsis* (*Arabidopsis thaliana*) Col-0 seeds were sown in nutrient soil after 3 days of vernalization at 4°C and then cultured at 22°C under 16 h light/8 h dark conditions for approximately 4 weeks. Rosettes were cut before flowering to prepare protoplasts for subcellular localization and BiFC assays.

Soybean (Glycine max) Williams82 seeds were placed on absorbent paper and placed in the dark at room temperature for three days to germinate. Seedlings that grew consistently in a sponge hole tray were cultured in 1/4 strength Hoagland’s solution for 14 days under 16 h light/8 h dark conditions at 25°C and 6000 lx with 80% relative humidity. Subsequently, 50 mM NaHCO_3_, 150 mM NaCl, 20% (w/v) PEG6000, 100 µM ABA, 100 µM MeJA, and 2 mM SA were added to Hoagland’s solution to simulate various abiotic stress conditions. Equal amounts of leaves and roots were collected for RNA isolation at 0, 3, 6, 12, and 24 h after treatment.

### Yeast two-hybrid assays

Yeast two-hybrid assays were performed according to the manufacturer’s protocol. Briefly, the full-length CDS of GmCaM (Glyma.19G121900) and 15 GmIQMs were cloned separately into pGADT7 and pGBKT7. The plasmids, AD-GmCaM and BD-GmIQMs, were co-transformed into AH109 yeast cells using the PEG/LiAC method. Interactions in yeast were tested on SD/-Trp/-Leu/-His and SD/-His-Trp-Leu-Ade plates containing 20 μg/mL x-α-gal. Cotransforming with the AD empty vector and BD-empty vector was used as a negative control, while AD-AtCAT2 and BD-AtIQM1 was used as a positive control (Lv et al., 2019).

For construction BD-GmIQM^LQ/VQ^ yeast expression vectors, LQ or VQ motif in CDS of five representative GmIQMs (GmIQM1a, 112-113aa LQ; GmIQM2a, 135-136aa LQ; GmIQM3a, 63-64aa VQ; GmIQM1e, 127-128aa LQ; GmIQM6a, 66-67aa LQ) was deleted using the PCR product fragment splicing method *in vitro*. Mutant protein sequences were cloned into the pGBKT7 vector. Thereafter, GmCaM binding to the mutant GmIQMs was analyzed using the yeast two-hybrid assay described above.

### Subcellular localization and BiFC assays

The protoplasmic transformation for both experiments was performed as previously described (Lv et al., 2019). For subcellular localization, the full-length CDS of 15 *IQM* genes in soybean Williams82 was cloned into the pGreenII-35S-GFP plant expression vector, respectively. Thereafter, pGreenII-35S-GFP-GmIQMs plasmids were transformed into protoplasts isolated from the leaves of 4-week-old *Arabidopsis* wild-type Col-0. After 16 h of incubation at 22°C, the GFP signal was observed using a Zeiss LSM800 confocal microscope at 488 nm absorption and 507 nm emission wavelengths. The pGreenII-35S-GFP empty vector was used as a negative control.

For the BiFC assays, the CDSs of GmIQM1d/GmIQM2c and GmCaM were cloned into binary pSAT1-nEYFP and pSAT1-cEYFP vectors, respectively. Thereafter, the pSAT1-nEYFP-GmIQM1d/GmIQM2c and pSAT1-cEYFP-GmCaM plasmids were transformed into protoplasts isolated from the leaves of 4-week-old Col-0. After 16 h of incubation at 22°C, the EYFP signal was observed using a Zeiss LSM800 confocal microscope at 488 nm absorption and 530 nm emission wavelengths. Co-transformation with nEYFP empty vector and cEYFP-GmCaM served as negative controls.

### RNA extraction and RT-qPCR analyses

RNA was isolated using the Eastep Super Total RNA Extraction Kit (Promega, LS1040) according to the manufacturer’s protocol. For the tissue-specific expression analysis, total RNA was extracted from roots (no nodule), cotyledons, stems, leaves, flowers, pods and nodules of Williams82. For the gene expression analysis under abiotic and biotic stress, total RNA was extracted from 14-days-old leaves and roots of Williams82, with or without treatment. RT-qPCR was performed as previously described (Bu et al., 2014; Lv et al., 2019), and first-strand complementary DNA (cDNA) synthesis was performed using the PrimeScript RT Reagent Kit (Takara, RR047A), according to the manufacturer’s instructions. cDNAs were used as templates for RT-qPCR with gene-specific primers. Primer sequences are listed in Supplemental **Table S2**. RT-qPCR was performed using 384-well plates with SYBR Premix Ex Taq II (Takara, RR820A) and a Roche Light Cycler 480 Real-Time PCR system. The soybean *tubulin* gene (Glyma.05G207500) was used as an internal control for mRNA (Li et al., 2017) and the relative expression levels of the genes were calculated using the 2^−CT^ method. Each sample was collected from five independent plants, independent biological assays were repeated three times, and similar results were obtained. Data from one representative replicate are shown. Values are means average from three technical measurements.

## Results

### Identification of *IQM* gene family in soybean genome

As revealed in previous studies, IQM proteins are plant-specific calcium-independent calmodulin-binding proteins that contain one IQ motif (Zhou et al., 2010). To identify the *IQM* gene family in soybean, we performed BLASTP analysis of the *Glycine max* genome *via* alignment with reported IQM proteins in *Arabidopsis*. Finally, 15 candidate *GmIQM* genes containing the IQ calmodulin-binding motifs were confirmed using conserved domain analysis. According to the order on chromosomes of *GmIQMs* and their homology relationship with *AtIQMs*, these 15 *GmIQM* genes were named *GmIQM1a-1f, GmIQM2a-2c, GmIQM3a-3c, GmIQM5*, and *GmIQM6a/6b*. The characteristics of each IQM protein, including location on chromosome, location coordinates, open reading frame (ORF) length, number of amino acids, molecular weight, theoretical pI (isoelectric points), and predicted subcellular localization, are listed in **Table 1**. According to these data, the protein sequences of the 15 soybean *IQM* genes did not significantly differ in length (467-661 aa) and molecular weight (52.29-74.58 kDa). Further, most proteins had high isoelectric points (pI 7.14-9.34), except GmIQM2a (pI 6.42) and GmIQM2b (pI 6.24). Almost all GmIQM proteins were predicted to localize in the nucleus, while only few were predicted to localize in the chloroplast or cytoplasm (**Table 1**).

**Table 1:** List of characteristics of 15 soybean *IQM* genes.

Name	Gene ID	Chr^a^.	Location Coordinates	ORF Length (bp)	Number of Amino Acid	Molecular Weight (Mw/Da)	Theoretical pI	WoLF PSORT^b^
*GmIQM1a*	Glyma.07G008300	Chr07	604283-609562	1500	499	56435.61	9.29	N, C
*GmIQM1b*	Glyma.08G191300	Chr08	15373388-15378072	1503	500	56490.62	9.13	N
*GmIQM1c*	Glyma.09G065000	Chr09	6326180-6330281	1587	528	59289.38	9.18	N
*GmIQM1d*	Glyma.15G025300	Chr15	2042923-2050342	1416	471	53821.58	9.02	N
*GmIQM1e*	Glyma.15G171000	Chr15	15602187-15606370	1584	527	59059.31	9.38	N
*GmIQM1f*	Glyma.17G053600	Chr17	4076879-4080511	1593	530	59547.40	9.45	N
*GmIQM2a*	Glyma.05G156600	Chr05	34903472-34907470	1971	656	73852.45	6.42	N
*GmIQM2b*	Glyma.08G114700	Chr08	8768854-8772973	1986	661	74581.42	6.24	N
*GmIQM2c*	Glyma.18G000400	Chr18	58631-61226	1680	559	63956.80	9.34	N
*GmIQM3a*	Glyma.10G067900	Chr10	6697335-6700998	1452	483	54230.08	8.91	N
*GmIQM3b*	Glyma.11G212100	Chr11	30470250-30473891	1425	474	53253.75	8.51	N
*GmIQM3c*	Glyma.19G193300	Chr19	45103820-45107114	1404	467	52289.03	8.95	CH, N
*GmIQM5*	Glyma.13G348500	Chr13	43866605-43870827	1527	508	57949.28	8.84	N
*GmIQM6a*	Glyma.02G168100	Chr02	25901408-25906110	1683	560	63999.54	7.14	N
*GmIQM6b*	Glyma.09G103600	Chr09	19197686-19201211	1788	595	67979.20	7.92	CH

a the chromosome in which the gene is located.

b N, nucleus; C, cytoplasm; CH, chloroplasts.

### Protein interaction analysis of GmIQMs with GmCaM

**Figure 1:**
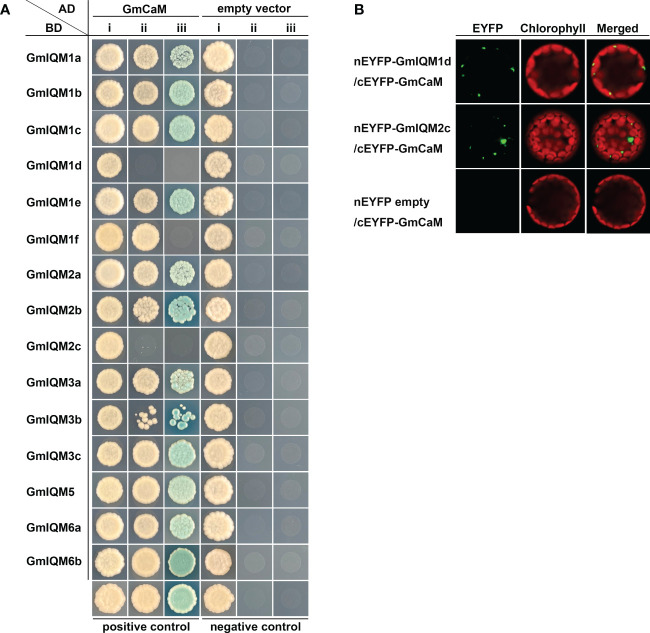
Interaction Between Gmiqms And Gmcam. **(A)** GmIQMs interact with GmCaM in yeast cells. pGADT7-GmCaM and pGBKT7-GmIQMs were co-transformed into AH109 yeast cells, which were grown on SD/-Trp-Leu (i), SD/-Trp-Leu-His (ii) and SD/-Trp-Leu-His-Ade containing 20 μg/ml x-α-gal (iii) for 3 days. Co-transformation with the AD empty vector and BD-empty vector served as negative controls. AD-AtCAT2 and BD-AtIQM1 were used as positive controls. **(B)** BiFC assays to detect the interaction between GmIQM1d/GmIQM2c and GmCaM. nEYFP-GmIQM1d/GmIQM2c and cEYFP-GmCaM plasmids were co-transformed into protoplasts isolated from the leaves of 4-week-old Col-0 plants. After 16 h of incubation, the EYFP signals were observed using a laser confocal microscope. nEYFP-empty vector and cEYFP-GmCaM were employed as negative controls.

Based on our previous study, IQMs can interact with CaMs in *Arabidopsis* and rice (Zhou et al., 2012; Fan et al, 2021 suggesting that binding to CaM is a common characteristic of IQM proteins. To further verify IQM in soybean, yeast two-hybrid experiments were carried out to determine whether IQM could also be combined with CaM in soybean. The results showed that only two vector combinations of AD-GmCaM and BD-GmIQM1d/2c could not grow on SD/-Trp/-Leu/-His triple dropout and SD/-Trp/-Leu/-Ade/-His quadruple dropout culture media (**Figure 1A**), indicating that most GmIQMs could interact with GmCaM in yeast cells, except GmIQM1d and GmIQM2c.

To further assess the interaction between GmIQM1d/GmIQM2c and GmCaM *in vivo*, an enhanced yellow fluorescent protein (EYFP)-based bimolecular fluorescence complementation (BiFC) assay was performed. Plasmids carrying nEYFP-GmIQM1d/GmIQM2c and cEYFP-GmCaM fused expression vectors were co-transformed into protoplasts of *Arabidopsis* Col-0. The co-transformation with the nEYFP empty vector and cEYFP-GmCaM was used as negative controls. Fluorescence signals were observed in the test group using a laser confocal microscope (**Figure 1B**).These results revealed that GmIQM1d/GmIQM2c interacts with GmCaM *in vivo* and a particular component of plant cells, which is nonexistent in yeast cells, and may be indispensable for their interactions. Overall, the reliability of soybean IQM proteins was confirmed using protein interaction assays.

### Phylogenetic and structural analyses of soybean *IQM* genes

**Figure 2:**
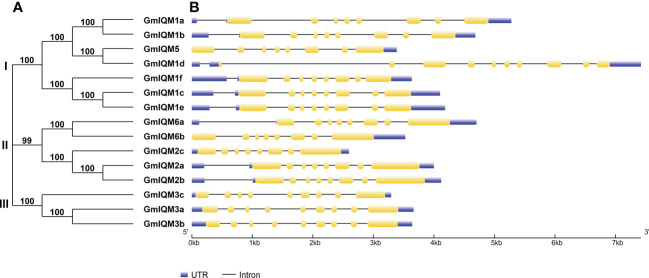
Phylogenetic relationships and gene structures of the *GmIQM* genes. **(A)** Unrooted phylogenetic tree of *GmIQMs*. The full-length amino acid sequences of 15 GmIQMs were aligned by ClustalW, and the unrooted phylogenetic tree was constructed using MEGA11 by the neighbor-joining method. The number of bootstrap values was 1000 replicates. **(B)** Exon/intron organization of soybean *IQM* genes. Yellow boxes represent exons, black lines represent introns, blue boxes represent Untranslated regions (UTRs). The sizes of exons and introns can be estimated using the scale at the bottom.

To understand the similarity and evolutionary relationship between GmIQM proteins, we constructed an unrooted phylogenetic tree of 15 soybean IQM protein sequences. Based on the results, all members of the soybean *IQM* gene family could be divided into three major subfamilies (I, II, and III; **Figure 2A**). Similar to the analysis results for *Arabidopsis* and rice, the IQM members in soybeans mainly belonged to subfamilies I and II. Notably, subfamily I had the most members, with seven genes, while subfamily III had the fewest IQM members, with three genes. Further, the most closely related members within each subfamily formed sister-gene pairs.

The diversity of gene structure is well known as an important foundation for gene family classification (Zhao et al., 2017; Mengarelli and Zanor, 2021; Zhou et al., 2021). To further detect the features of each IQM subfamily in the soybean genome, the exon/intron organization of *GmIQM* genes was generated (**Figure 2B**). The structure chart revealed that most soybean *IQM* genes (eleven/fifteen) contained eight exons, except *GmIQM1d*, which contained ten exons, and three members of subfamily III (*GmIQM3a*, *3b*, and *3c*), which had nine exons. The relatively uniform exon numbers indicate the structural conservation of *GmIQM*.

The most closely related *GmIQM* gene pairs in the same subfamily shared similar gene structures, both in exon distribution and intron length. Nevertheless, one pair (*GmIQM1d*/*GmIQM5*) displayed an obvious discrepancy (**Figure 2B**); compared with *GmIQM5*, *GmIQM1d* had two extra short coding sequences in front of the first exon, which may be due to intron gain events during the long evolutionary period.

**Figure 3:**
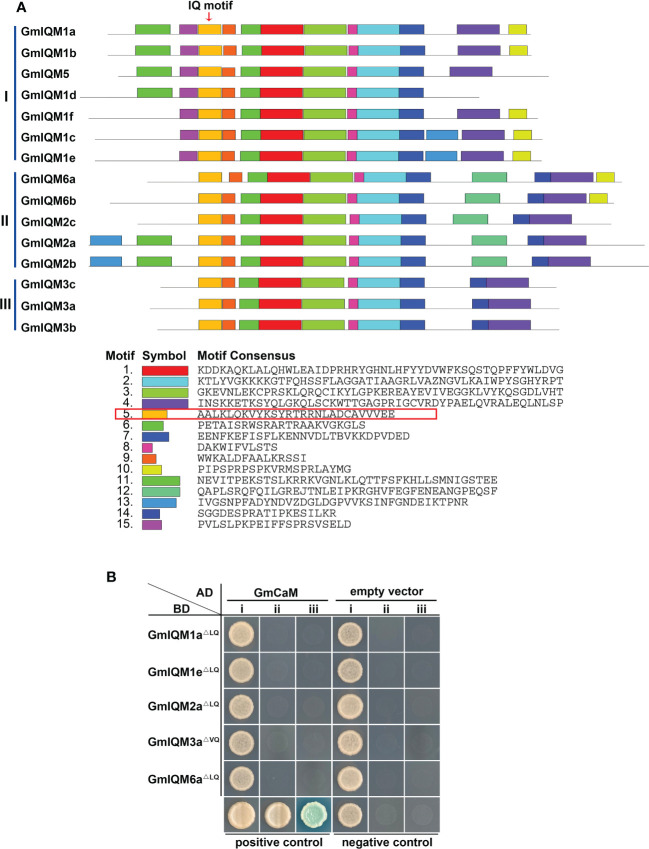
Conserved motif in GmIQM proteins. **(A)** Motif distribution in the IQM proteins of soybean. Motifs of the GmIQM proteins were identified using the MEME online program. The maximum number of motifs was set as 15. Fifteen motifs were represented by different colors; each motif consensus sequence is listed below, red box represents the amino acid sequence of the IQ motif, arrow above the diagram represents the location of the IQ motif. **(B)** Functional validation of the IQ motif in GmIQM proteins. AD-GmCaM and BD-GmIQM^LQ/VQ^ were co-transformed into yeast cell AH109 grown on SD/-Trp-Leu (i), SD/-Trp-Leu-His (ii), and SD/-Trp-Leu-His-Ade containing 20 μg/ml x-α-gal (iii) for 3 days. Co-transformation with the AD empty vector and BD-empty vector was performed for use as the negative controls. AD-AtCAT2 and BD-AtIQM1 served as the positive controls.

The conserved domains or motifs of 15 *GmIQM* genes were examined using the MEME database. Fifteen potentially conserved motifs were identified (**Figure 3A**; **Figure S1**, and **S2**). Motif 5 was annotated to encode the IQ motif based on a data search of Pfamscan and SMART. The distribution of motifs in 15 GmIQMs is consistent with the gene clustering that

Most closely gene pairs in the same subfamilies had identical motifs, except *GmIQM1d*/*GmIQM5* (**Figure 3A**), suggesting functional similarities or redundancies among these GmIQM proteins. Further, the 15 GmIQM proteins had common motifs (motifs 1, 2, 3, 5, 6, 7, 8, and 9); however, some motifs were recognized to be specific to certain subfamilies. For example, motifs 12 and 15 are unique to subfamilies II and I, respectively. Thus, variations in these specific motifs may cause functional differentiation of the IQM proteins in soybean.

The IQ motif is key to the interaction between CaM and CaMBP (Rhoads and Friedberg, 1997). To determine whether the combination of GmCaM and GmIQMs is dependent on the IQ motif, we constructed a BD vector with the soybean IQM protein sequence deleted 6Q (LQ/VQ) motif. Thereafter, AD-GmCaM and BD-^LQ/VQ^ were co-transformed into yeast cell AH109. As all transformants did not grow on the SD/-Trp/-Leu/-Ade/-His quadruple dropout culture medium (**Figure 3B**), the IQ motif was identified to be necessary for functional GmIQM and its conservation in different species.

### Chromosomal locations and gene duplication

**Figure 4:**
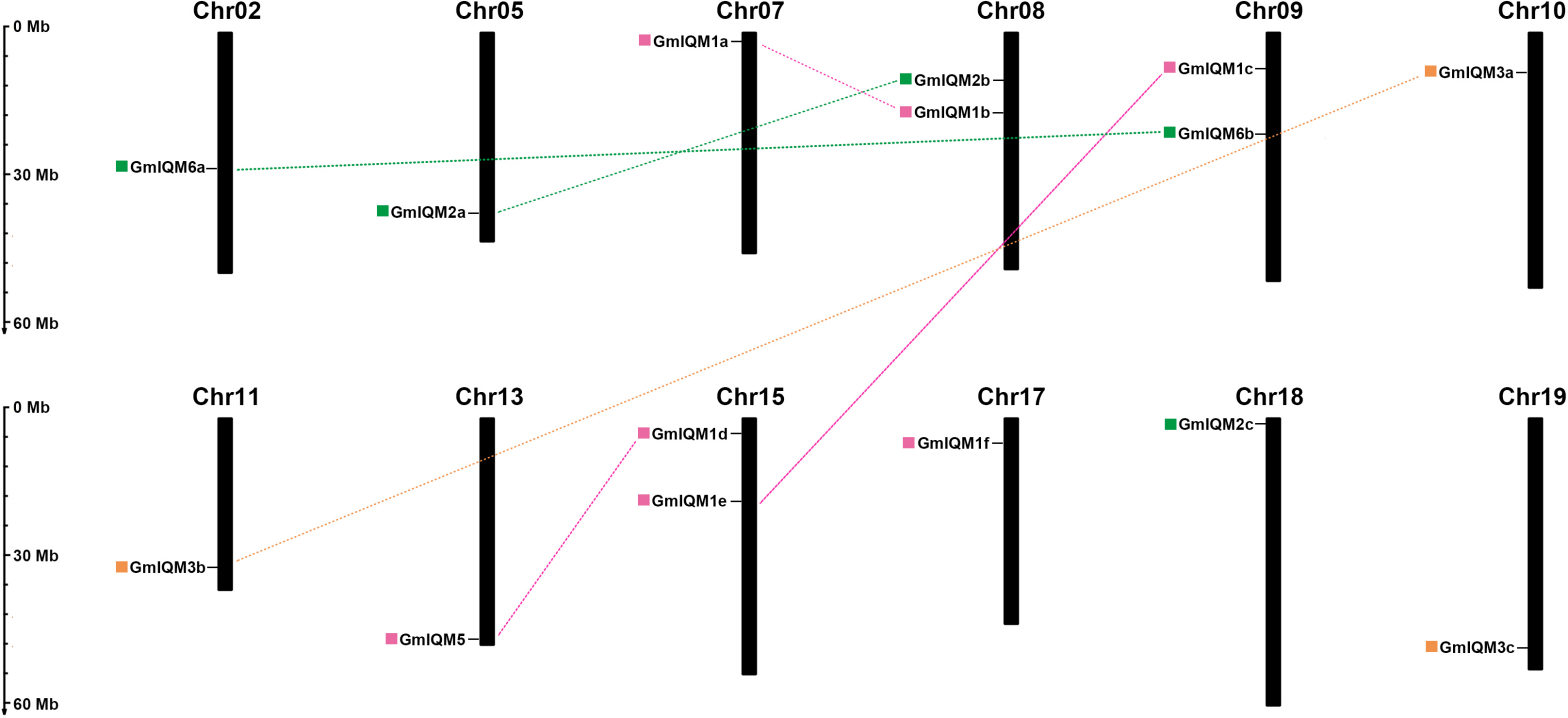
Chromosomal locations and segmental duplication of soybean *IQM* genes.The fifteen *IQM* genes are widely mapped to 12 of the 20 chromosomes in soybean. Chromosome numbers are located at the top of each vertical bar. The duplicated paralogous pairs of *GmIQM* gene are connected with dotted lines of the same color. The colored boxes in front of the gene names represent different subfamilies (pink, subfamily I; green, subfamily II; orange, subfamily III). Scale on the left represents the chromosome length.

To accurately understand the orientation of the 15 soybean *IQM* genes on each chromosome, a chromosomal map we constructed based on the location information retrieved from the soybean database. The distributions of these genes on chromosomes appeared to be wide but unbalanced. Fifteen *GmIQM* genes were mapped on 12 of the 20 soybean chromosomes. Nine chromosomes, including chromosomes 2, 5, 7, 10, 11, 13, 17, 18, and 19, contained only one *IQM* gene. Three chromosomes, including chromosomes 8, 9, and 15, had two *IQM* genes, while the remaining eight soybean chromosomes did not contain any *IQM* gene (**Figure 4**). This biased distribution pattern of *IQM* genes has been observed in *Arabidopsis* and rice genomes (Zhou et al, 2010; Fan et al, 2021). Although two *IQM* genes were present on chromosomes 8, 9, and 15, however, there was no clustering from *GmIQM* genes on the same chromosomes, suggesting that the expansion of *IQM* family genes in soybean may not have been produced from tandem duplications.

Gene duplication is considered an important source of biological evolution. There are three main types of gene duplication: segmental duplication, tandem duplication, and transposition (Sankoff, 2001; Flagel and Wendel, 2009; Panchy et al., 2016). Among these duplication types, segmental duplication is a major contributor to the amplification of many gene families (Cannon et al., 2004; Zhu et al., 2020; Li et al., 2021; Wang M, et al., 2021; Zhao et al., 2021);. Herein, gene duplication events were examined to further understand the expansion mechanism of the *IQM* family in soybean. According to the data of synteny (*Glycine* recent duplication) from SoyBase browser, we found six pairs of

*GmIQM* genes located in duplicated blocks, indicating that these genes were generated by segmental duplication. The remaining three genes (*GmIQM1f*, *2c*, and *3c*) lacked duplicated pairs in their corresponding synteny blocks (**Figure 4** and **Table 2**), which aligns with the results of clustering analysis. We determined whether tandem duplication also played a role in adjacent *GmIQM* genes on the same chromosome. It has been reported that a pair of genes is separated by three or fewer genes within a 100-kb region on a chromosome, this may be due to tandem duplication (Wang et al., 2010; Feng et al., 2014; Wang X, et al., 2021). According to this criterion, no pair was generated by tandem duplication of the soybean *IQM* genes. Therefore, segmental duplication was identified to contribute significantly to the expansion of the *IQM* gene family in soybean.

**Table 2:** Divergence between paralogous *IQM* gene pairs in soybean.

No.	Group	Paralogous pairs	Ka	Ks	Ka/Ks	Duplication date (MY)	Duplicate type^a^
1	Ia	*GmIQM1a*-*GmIQM1b*	0.037	0.107	0.344	8.734	S
2	Ia	*GmIQM5*-*GmIQM1d*	0.061	0.179	0.342	14.674	S
3	Ib	*GmIQM1c*-*GmIQM1e*	0.034	0.153	0.224	12.501	S
4	IIa	*GmIQM2a*-*GmIQM2b*	0.028	0.103	0.270	8.461	S
5	IIa	*GmIQM6a*-*GmIQM6b*	0.028	0.089	0.314	7.263	S
6	IIIa	*GmIQM3a*-*GmIQM3b*	0.035	0.158	0.222	12.987	S

a S, segmental duplication.

To understand the evolutionary selection of duplicated *GmIQM* genes, we calculated the Ka/Ks ratio (substitution ratio of non-synonymous/synonymous) for each pair of duplicated *GmIQM* genes. In general, Ka/Ks = 1 indicates that both genes drift neutrally, Ka/Ks>1 indicates accelerated evolution with positive selection, and Ka/Ks<1 indicates a functional constraint with negative or purifying selection of the genes (Hurst, 2002; Nekrutenko et al., 2002; Feng et al., 2014; Ma et al., 2014). The Ka/Ks ratios from all duplicated gene pairs were found to be less than 0.4 (**Table 2**), suggesting that the evolution of the soybean *IQM* gene family was mainly influenced by negative or purifying selection, thereby limiting the functional differentiation of duplicated genes. Based on the divergence rate of 6.1×10^-9^ synonymous mutations per site per year (Lynch and Conery, 2000; Zhang et al., 2011; Chen et al., 2014; Hyun et al., 2014; Meng et al., 2015; Zhang et al., 2019), the duplication of these paralogous pairs was estimated to occur between 7.263 and 14.674 million years ago (Mya) (**Table 2**).

### Phylogenetic comparison of the *IQM* genes in various species

**Figure 5:**
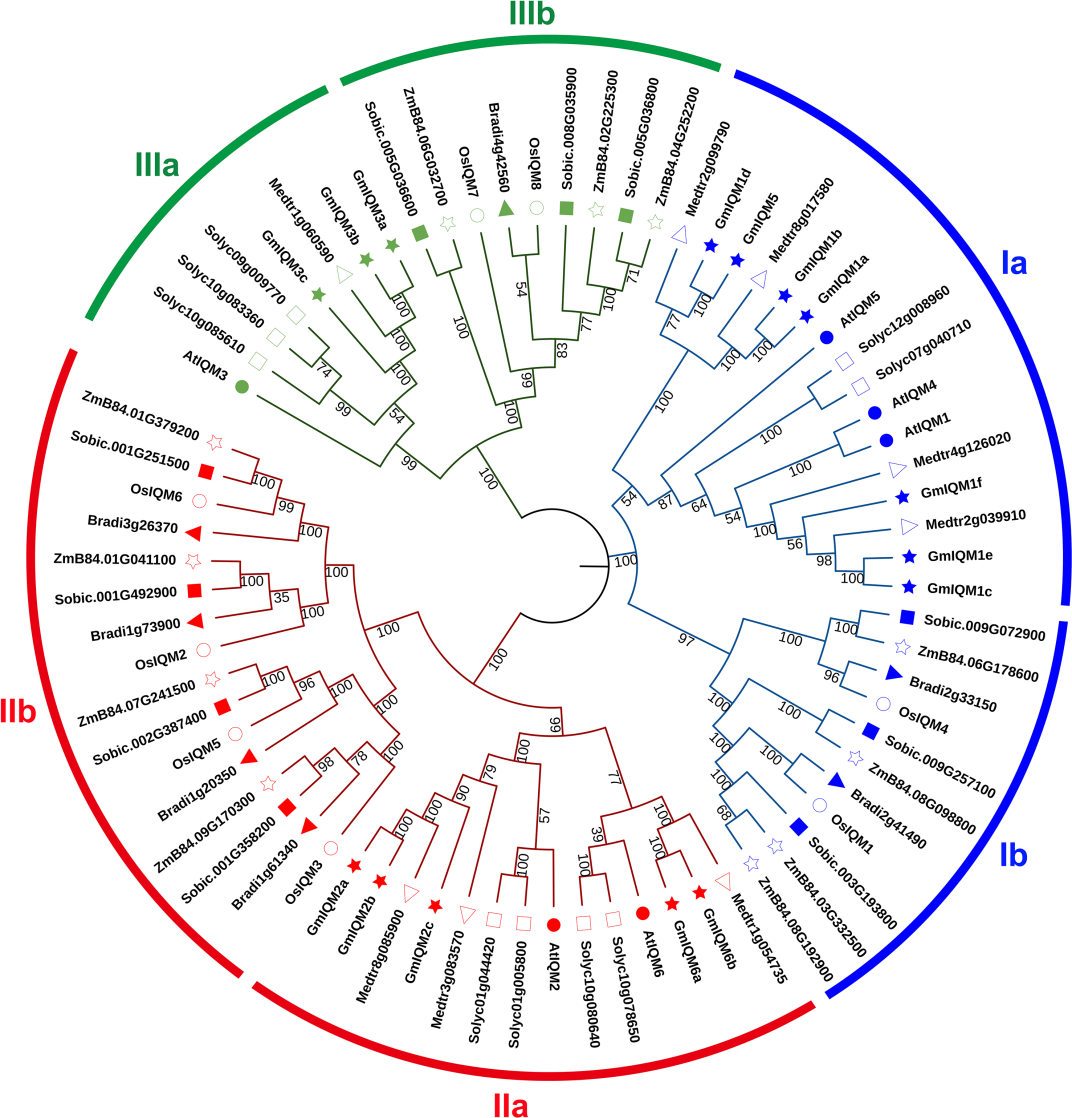
Phylogenetic relationships of *IQMs* in eight species.The full-length amino acid sequences of 74 IQMs were aligned using ClustalW, and the unrooted phylogenetic tree was constructed using MEGA11 by the neighbor-joining method. The number of bootstrap values was 1000 replicates. At, Arabidopsis; Gm, soybean; Medtr, alfalfa; Solyc, tomato; Os, rice; ZmB84, maize; Sobic, sorghum; Bradi, *Brachypodium distachyon*. Blue, subfamily I; red, subfamily II; green, subfamily III. The shapes preceding the gene numbers represent different species.

Seventy-four putative IQM protein sequences from eight species, including four dicotyledons, *Glycine max* (15)*, Arabidopsis thaliana* (6)*, Solanum lycopersicum* (9)*, Medicago sativa* (8); and four monocotyledons, *Oryza sativa* (8)*, Zea mays* (11)*, Sorghum bicolor* (10), and *Brachypodium distachyon* (7), were analyzed using the neighbor-

joining (NJ) method to further assess the phylogenetic relationship of *IQMs* in plants. All *IQMs* were found to cluster into three distinct subfamilies (I, II, and III) (**Figure 5**). This result aligns with the classification of *GmIQM* genes; subfamilies I and II had the most members (77%) in the combined phylogenetic tree, while subfamily III had the fewest genes (23%). Each subfamily was further divided into two subgroups (a and b) of dicots and monocots (**Figure 5**), indicating that significant differences in the structure and function of *IQM* genes between dicots and monocots.

### Subcellular localization of GmIQMs

**Figure 6:**
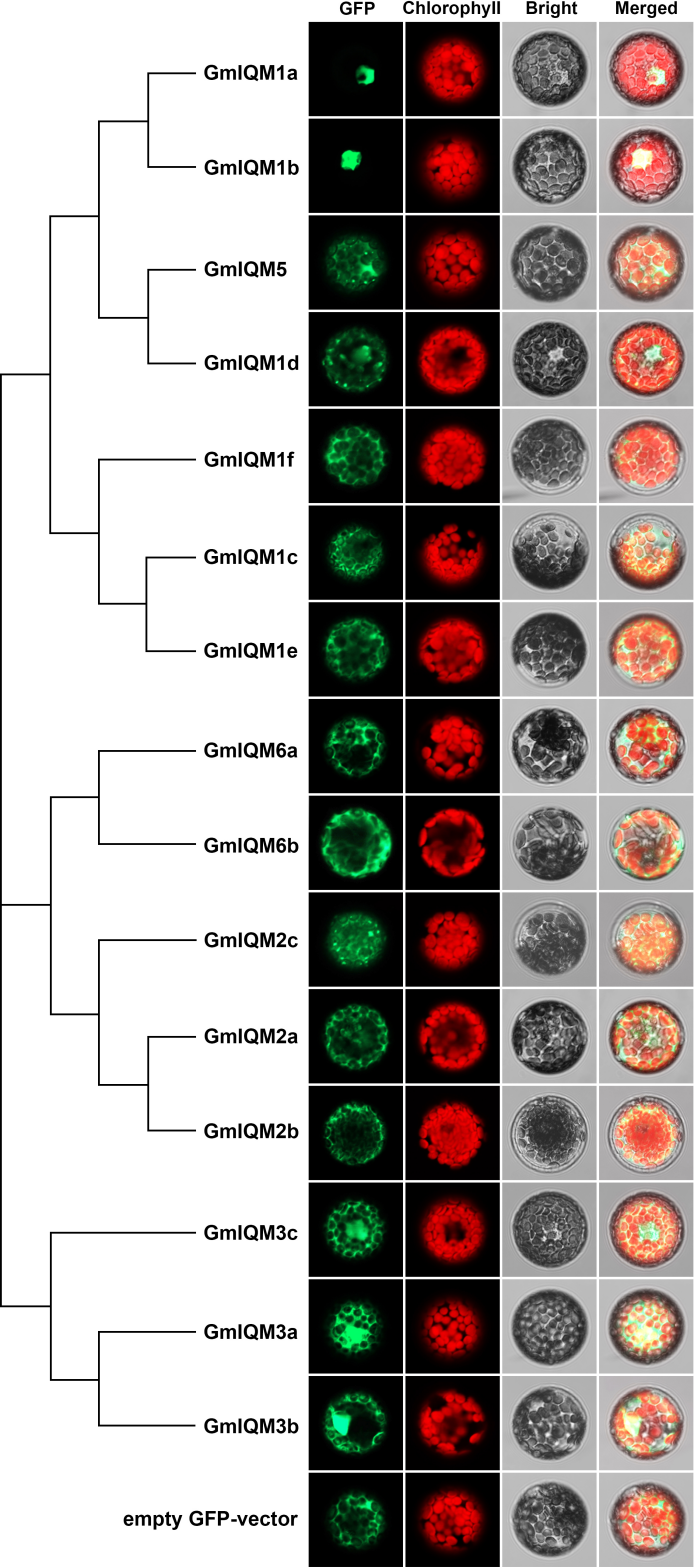
Subcellular localization of fifteen GmIQM proteins in *Arabidopsis* protoplasts.The plasmids containing pGreenII-35S-GFP-GmIQMs vector were transformed into protoplasts isolated from the leaves of 4-week-old Col-0 plants. After 16 h of incubation, the GFP signals were observed using a laser confocal microscope. Transformed pGreenII-35S-GFP empty vector was employed as the negative control. GFP, green fluorescent protein; Chlorophyll, chlorophyll auto-fluorescence; Bright, bright field; Merged, merged GFP, chlorophyll and bright.

The subcellular localization of 15 soybean IQM proteins was determined to further confirm their functional sites in cells. The full-length CDS sequence of each GmIQM was cloned and constructed into a pGreenII-35S-GFP vector, and different plasmids were transformed into protoplasts of *Arabidopsis* wild-type Col-0. After incubation, the GFP signal was observed under a confocal microscope (**Figure 6**). The results showed that GmIQM1a and GmIQM1b were only located in the nucleus; GmIQM3a, 3b, and 3c were located in the nucleus and cytoplasm; and the remaining IQMs were mainly localized in the cytoplasm. These location patterns matched the clustering results, members of the closest relative clade had the same localization in the cell, indicating that these genes may have the same or similar functions. Further, the practical localization of these genes did not completely match the prediction by the Wolf PSORT programs.

### Tissue-specific expression of *GmIQM* genes

**Figure 7:**
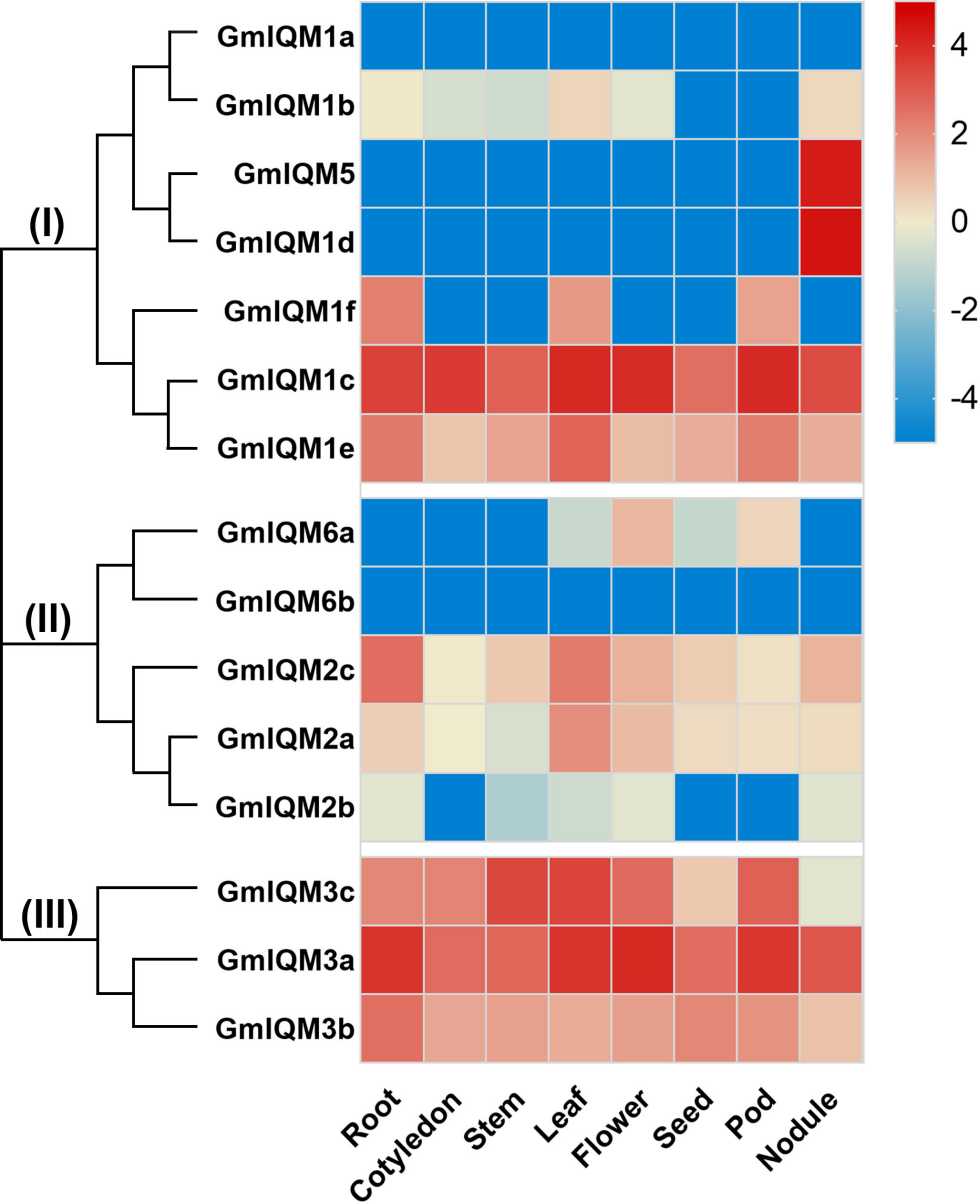
Heat map of real-time quantitative PCR analysis results of *GmIQM* in eight tissues.Total RNA was extracted from roots, cotyledons, stems, leaves, flowers, pods, and nodules. Each sample was collected from five independent plants, independent biological assays were repeated three times, and similar results were obtained. Data from one representative replicate are shown. Values are means average from three technical measurements. Color scale at the right side of the image represents log10 of the gene expression values. Red indicates high expression level and blue indicates low expression level.

Every stage of plant growth and development is closely regulated by a large number of genes. To understand the potential role of soybean *IQM* genes in the plant life cycle, quantitative polymerase chain reaction (qRT-PCR) was performed to determine the expression levels of *IQM* genes in soybean roots (no nodule), cotyledons, stems, leaves, flowers, seeds, pods, and nodules respectively. As depicted in the heat map, soybean *IQM* genes from the same subfamily or the most closely related paralogous gene pairs had similar gene expression patterns. For example,

Three members of subfamily III (*GmIQM3a*, *3b*, and *3c*) were significantly expressed in eight tissues. However, in subfamily I, only two genes, *GmIQM1c* and *GmIQM1e*, which are sister pairs in the closest clade, had universal expression, and the remaining genes (*GmIQM1a*, *GmIQM1b*, *GmIQM5*, *GmIQM1d*, and *GmIQM1f*) were not expressed in most tissues (**Figure 7**). Notably, some *GmIQM* genes have tissue-specific expression characteristics, such as *GmIQM5* and *GmIQM1d*, which had markedly high transcript abundance in nodules but were not expressed in other tissues, and *IQM6a*, which only had a slight expression in flowers (**Figure 7**), suggesting that these genes might have vital functions at specific stages of soybean development.

### Analysis of *cis*-acting elements of *GmIQM* gene promoters

**Figure 8:**
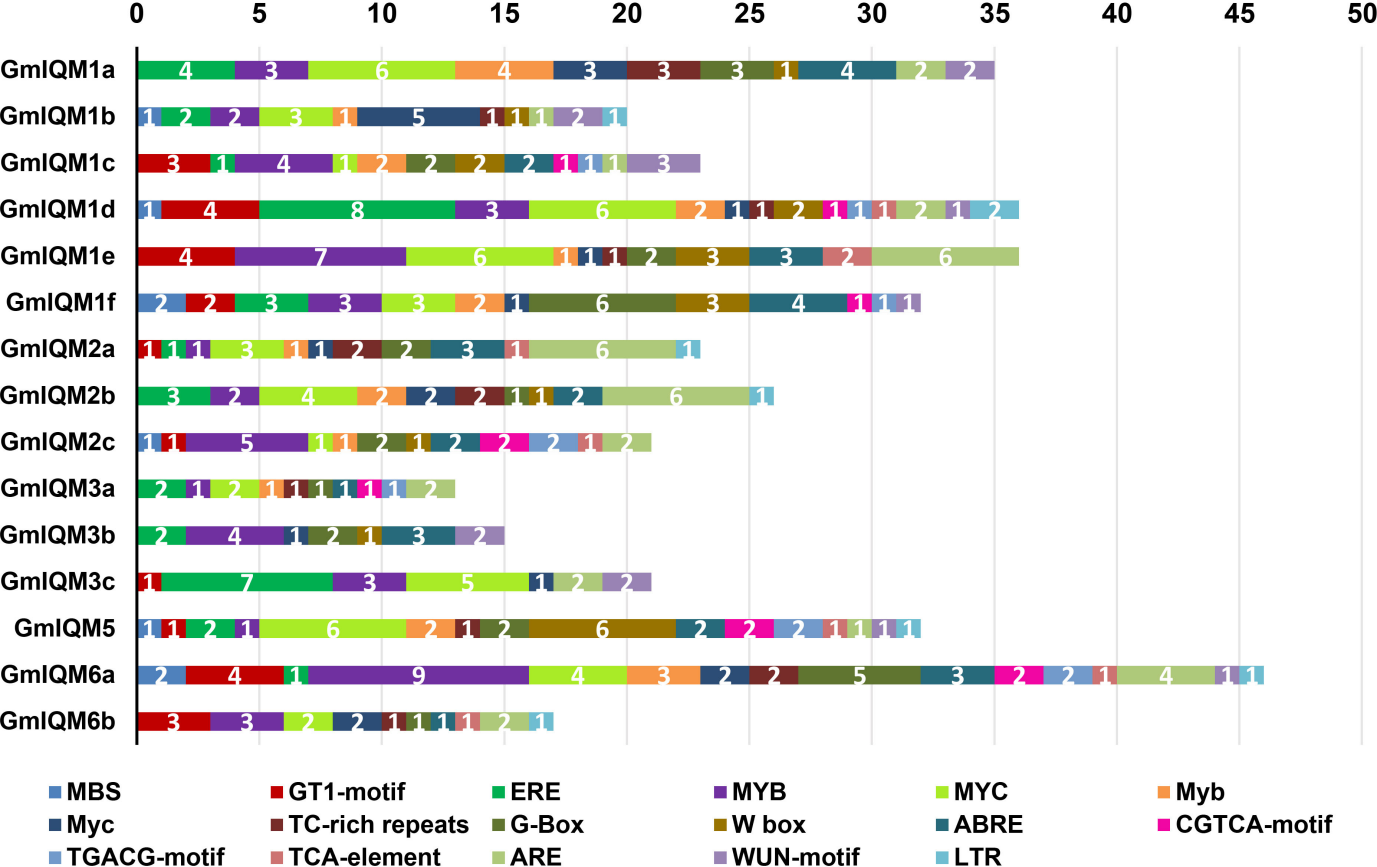
Putative *cis*-acting elements of the *IQM* promoters predicted by PlantCARE.The different *cis*-elements are indicated by different colors. The numbers on the box represent the count of *cis*-elements in the promoter.

The type and number of *cis*-acting elements in gene promoters are well known to largely determine gene function. To explore the potential role of soybean *IQM* genes, we analyzed the *GmIQM* gene promoter sequences within 2 kb upstream of the start codon using the PlantCARE database. In addition to the core elements, TATA-box and CAAT-box, various stress and hormone response elements were found in the promoter of each soybean *IQM* gene, such as MBS, GT1, ERE, MYB, MYC (drought, high salt, low temperature, etc.), ABRE (ABA), CGTCA-motif, TGACG-motif (MeJA), and TCA-element (SA) (**Figure 8**). Therefore, *GmIQMs* could play a role in the response of soybean to biotic and abiotic stresses. However, the number and distribution range of abiotic response elements were significantly higher than those of MeJA and SA stress-related elements, especially MYB and MYC, which existed in almost all soybean *IQM* promoters. Thus, the soybean *IQM* gene family may be more important for abiotic stress response than biotic defense.

### Effects of abiotic and biotic stress on *GmIQM* gene expression

**Figure 9:**
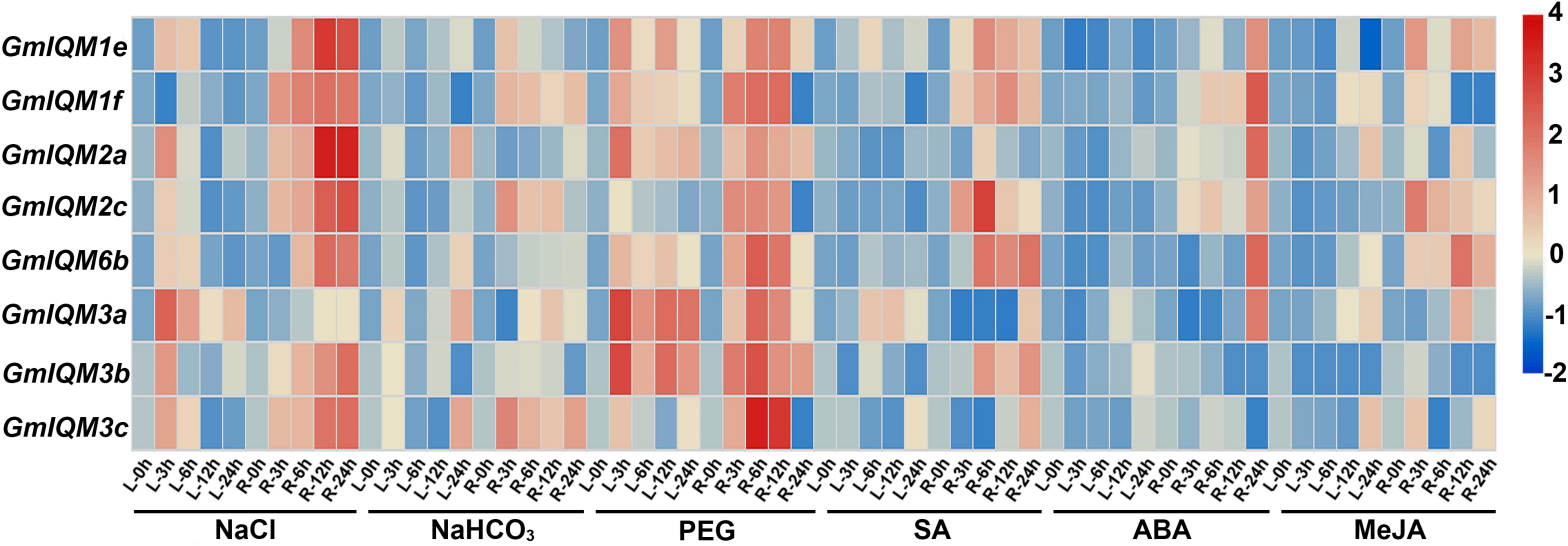
Heat map of the expression of *GmIQMs* in leaves and roots under different treatments.NaCl, NaHCO3, PEG, SA, ABA, and MeJA were used to treat soybean. Total RNA was then extracted from the roots and leaves at indicated times. Each sample was collected from five independent plants, independent biological assays were repeated three times, and similar results were obtained. Data from one representative replicate are shown. Values are means average from three technical measurements. Color scale at the right side of the image represents log10 of the gene expression values. Red indicates high expression level and blue indicates low expression level. L, leaves; and R, roots.

According to the analysis of *cis*-acting elements in promoters, *IQM* genes are most likely regulated by abiotic stresses and hormonal stimuli in soybean. To further confirm the function of the *GmIQM* genes, soybean wild-type Willims82 was treated with different stressors, including NaCl, NaHCO_3_, PEG, SA, ABA, and MeJA, to simulate saline, alkaline, drought, and biological stress conditions. After treatment, the expression levels of eight representative *IQM* genes in soybean leaves and roots were determined using qRT-PCR. Gene expression profiling revealed that these *IQM* genes were strongly induced by NaCl and PEG, both in the leaves and roots (**Figure 9**). Thus, these *IQM* genes play an important role in the response to high salinity and drought conditions in soybean. These genes were also upregulated by NaHCO_3_, SA, ABA, and MeJA; however, compared with NaCl and PEG, the induction degree of their expression levels was relatively low under these stresses, and the transcriptional change mainly occurred in the roots, and not in the leaves (**Figure 9**). These results indicated that *IQM* family genes also function in alkali and biological stress responses to some extent.

## Discussion

As plant-specific calcium signaling components, *IQM* genes have been postulated to play a critical role in the crosstalk between multiple signaling pathways in the context of plant growth and development (Lv et al., 2019; Zhang et al., 2022). In our previous studies, we conducted a systematic and comprehensive analysis of *IQM* family genes in *Arabidopsis* and rice (Zhou et al., 2010; Fan et al., 2021). However, the number and functions of *IQM* genes in other species remain unclear. Herein, we identified 15 *IQM* genes in soybean, and employed bioinformatics to analyze the soybean *IQM* family genes at the whole-genome level.

Compared to *Arabidopsis* (six members) and rice (eight members), the *IQM* gene family in soybean is by far the largest. In fact, the number of *IQM* genes in soybean is 2.5-fold that of *Arabidopsis*. This doubling of the gene number has been observed in many other soybean gene families. For example, *IQD*, another IQ-containing calmodulin binding protein family, has 67 members in soybean, which is more than twice that in *Arabidopsis* (33 members) (Abel et al., 2005; Feng et al., 2014). The basic leucine zipper (b-ZIP) transcription factor family contains 78 members in *Arabidopsis* and 160 members in soybean (Jakoby et al., 2002; Dröge-Laser et al., 2018; Zhang et al., 2018). The genome-wide duplication (WGD) events in soybean evolutionary history may explain this phenomenon. WGD is very common in plants, leading to double gene copies in the genome. The functional divergence of duplicate gene pairs is the source of new genes. Soybean has been reported to experience at least two WGD events, approximately 59 and 13 million years ago, resulting in a highly duplicated genome with nearly 75% of the genes present in multiple copies (Schmutz et al., 2010). Therefore, a greater expansion of *IQM* genes may have occurred in the soybean genome than in other species. According to our analysis, six paralogous pairs were present among the 15 *GmIQM* genes, and all six pairs were caused by segmental duplication, but not tandem duplication (**Table 2**). Such findings indicate that segmental duplication played a more important role in the long evolution of soybean *IQM* genes. The Ka/Ks ratio was less than 1 for all 6 pairs of duplicated genes, indicating that these gene pairs were subjected to purifying selection (Hurst, 2002). The calculations also revealed that the duplication events of the 6 paralogous pairs in the soybean *IQM* family occurred between 7.26 and 14.27Mya, which was during the recent WGD of soybean. This date predates the duplication of the *IQM* gene pairs in rice (11.9-19.8 Mya; Fan et al., 2021).

By comparing the predicted *IQM* sequences from multiple species, the *IQM* genes in three different subfamilies were further divided into two groups: dicots(*Arabidopsis*, soybean, alfalfa, and tomato) and monocots (rice, maize, sorghum, and *Brachypodium distachyon*), indicating that the *IQM* gene sequences in monocots and dicots are conserved and significantly different. In general, two genes from ortholog pairs in phylogenetic trees are often considered to have a common ancestor and may have similar functions (Fan et al., 2021). In our study, most of the closest ortholog gene pairs in monocotyledons comprised maize/sorghum and rice/*Brachypodium distachyon*, indicating that *IQM* genes from the four monocots species are more closely related to each other than to those from the same species in the three subfamilies. However, for dicotyledons, the closest gene pair were paralogous gene from the same species. Thus, the *IQM* family genes of dicots are more closely conserved than those of monocots. Nevertheless, the *IQM* genes of the same species were not clustered in the closest proximity within the dicot sub-branch, but were dispersed among different dicot species, indicating that there was a common *IQM* ancestor in different dicots. Further, the closestgene pairs were not found between monocotyledons and dicotyledons, indicating that the ancestral *IQM* genes did not exist before the dicot-monocot divergence.

According to a previous study, IQM is a calcium-independent calmodulin-binding protein family that contains one IQ motif with a sequence composition of IQxxxRGxxxR or its more relaxed version, [ILV]QxxxRxxxx [R, K] (Feng et al., 2014). The IQ motifs of 15 soybean *IQM* genes are listed in **Table S1**. The IQ motif in soybean *IQM* genes was [ILV]Qxxx[K/R]xxxxR, which is more inclined to its relaxed version; however, the amino acid sequence at position 6 of most IQ motifs was K instead of R, except *GmIQM3a*, *GmIQM 3b*, and *GmIQM 3c*. IQM can bind to CaMs through the IQ motif in *Arabidopsis* and rice, indicating that the IQ motif is key for the *IQM* gene to play a role in calcium signaling. To demonstrate the reliability of the predicted soybean IQ motif, we validated the role of the IQ motif using yeast two-hybrid and bimolecular fluorescence complementation (BiFC) experiments. Based on our results, the 15 soybean IQMs could bind to GmCaM protein in yeast or plant cells. However, the GmIQM protein-deleted IQ motif completely lost its ability to bind GmCaM, suggesting that the role of the IQ motif is conserved and crucial in *Arabidopsis*, rice and soybean. These results also indicate that the soybean *IQM* genes identified in this study were reliable.

Soybean *IQM* genes were divided into three subfamilies according to their sequences. This clustering is supported by the results of structural analysis of *IQM* genes and proteins. Members in the same groups were found to be highly conserved in terms of intron length, exon number, and motif distribution. For example, all five members of subfamily II contained eight exons, while three members of subfamily III contained nine exons (**Figure 2**),and the motifs 15 and 12 were unique to subfamilies I and II, respectively (**Figure 3**). The differentiation of gene sequences leads to diversity in gene function. This was confirmed by the isoelectric point (pI) values, subcellular localization, and gene expression analysis. The pI values of 15 *IQM* genes ranged from 6.24 to 9.45; however, the paralogous pairs or genes in the same subfamily share very similar parameters (**Table 1**). For example, the pI values of *GmIQM1a/1b* and *GmIQM2a/2b* were 9.29/9.13 and 6.42/6.24, respectively. The location patterns of 15 *GmIQM* genes matched the clustering results, with the closest related clade members having the same intracellular localization, such as *GmIQM1a* and *GmIQM1b* were localized only in the nucleus, whereas *GmIQM3a* and *GmIQM3b* in both the nucleus and cytoplasm, indicating that genes adjacent to each other on the phylogenetic tree may have the same or similar functions (**Figure 6**). In addition, the real-time PCR also revealed that the paralogous pairs had similar tissue expression patterns (**Figure 7**). These results provide strong evidence to support the clustering of soybean *IQM* family genes and imply the complex functions of *GmIQM* genes.

Members of a single-gene family may have different biological functions. In *Arabidopsis*, AtIQM1 modulates stomatal movement by affecting ROS levels (Zhou et al., 2012) and participates in plant disease response signaling by promoting the synthesis of JA (Lv et al., 2019); AtIQM4 is positively involved in seed dormancy and germination by regulating ABA content (Zhou et al., 2018); AtIQM5 regulates flowering by modulating the juvenile-to-adult transition (Gong et al., 2017) and interacts with IAAs, a key repressor of auxin signaling, to promote lateral root and callus formation (Zhang et al., 2022). These studies revealed multiple roles of *AtIQM* genes throughout the plant life cycle. However, the functions of the *IQM* genes in soybean have not been reported. To preliminarily explore the potential function of the GmIQMs, we conducted an expression analysis of the 15 *GmIQM* genes in different tissues or organs using qRT-PCR. The absence of *GmIQM1a* and *GmIQM6b* in eight different tissues indicates that these genes may be pseudogenes or may be expressed at a specific time point in the course of soybean life or in response to an external stimulus. *GmIQM1c*, *GmIQM1e*, *GmIQM3a*, *GmIQM3b*, and *GmIQM3c* were highly abundant in various tissues, suggesting that these genes may play multiple roles in soybean development. Finally, *GmIQM1d* and *GmIQM5* were specifically and markedly expressed only in nodules, suggesting their important functions in nitrogen fixation or communication between plants and rhizosphere microorganisms. *GmIQM6a* was hardly expressed in other tissues, except flowers, indicating that this gene may only play a specific role during soybean flowering. These results imply that widely expressed genes may play a role in multiple aspects of the entire plant life course, and other *IQM* genes with little or no expression may be activated in response to certain environmental stimuli with irritability.

After treatment with NaCl, NaHCO_3_, PGE, SA, ABA, and MeJA, the expression levels of representative *GmIQM* genes (*GmIQM1e, 1f, 2a, 2c, 3a, 3b, 3c, 6b*) were significantly induced, especially with PEG and NaCl, indicating that *GmIQMs* may play an important role in salt and drought resistance. However, our results do not exclude the role of soybean *IQM* genes in the response to alkali and other abiotic stresses, and disease and insect invasion in soybean. The promoter analysis also showed that most soybean *IQM* genes contained multiple *cis*-acting elements related to abiotic and biotic stresses, but there were fewer related to biotic stresses. The results of the two parts confirmed each other, which further proved the credibility of the *GmIQMs* function in abiotic and biotic stress responses. There are six *IQM* genes, including *GmIQM1a, 1b, 1d, 2b, GmIQM5* and *6a* that are not expressed or expressed at very low levels in soybean leaves and roots, and whose transcript levels remain unchanged under various treatments (results are not shown), indicating that these genes may function only transiently at a particular time point.

In conclusion, fifteen *IQM* genes were identified in soybean. These genes have a variety of functions based on their structures, especially in response to abiotic stress (salt and drought) reactions in soybean, in which they may play an important role. Our systematic analysis of *IQM* family genes provides a theoretical foundation and a clear direction for subsequent in-depth research on the biological functions of each soybean *IQM* genes.

## Data availability statement

The datasets presented in this article are not readily available because. Requests to access the datasets should be directed to changentian@aliyun.com.

## Author contributions

CT, and TL designed the experiments, supervised the study, and managed the projects. TL performed most of the research and drafted manuscript. QL performed bioinformatics analysis and charting. HX participated in some of the experiments. TF, YZ, JW and CT analyzed and discussed the results. All authors contributed to the article and approved the submitted the version.

## References

[B1] Abel S. Savchenko T. Levy M. (2005). Genome-wide comparative analysis of the IQD gene families in arabidopsis thaliana and oryza sativa. BMC evolutionary Biol. 5, 72. doi: 10.1186/1471-2148-5-72 PMC1368998 16368012

[B2] Aldon D. Mbengue M. Mazars C. Galaud J. P. (2018). Calcium signalling in plant biotic interactions. Int. J. Mol. Sci. 19 (3), 665. doi: 10.3390/ijms19030665 29495448 PMC5877526

[B3] Ali E. Raza M. A. Cai M. Hussain N. Shahzad A. N. Hussain M. . (2020). Calmodulin-binding transcription activator (CAMTA) genes family: Genome-wide survey and phylogenetic analysis in flax (Linum usitatissimum). PloS One 15 (7), e0236454. doi: 10.1371/journal.pone.0236454 32702710 PMC7377914

[B4] Bouché N. Scharlat A. Snedden W. Bouchez D. Fromm H. (2002). A novel family of calmodulin-binding transcription activators in multicellular organisms. J. Biol. Chem. 277 (24), 21851–21861. doi: 10.1074/jbc.M200268200 11925432

[B5] Bouché N. Yellin A. Snedden W. A. Fromm H. (2005). Plant-specific calmodulin-binding proteins. Annu. Rev. Plant Biol. 56, 435–466. doi: 10.1146/annurev.arplant.56.032604.144224 15862103

[B6] Bu Q. Lv T. Shen H. Luong P. Wang J. Wang Z. . (2014). Regulation of drought tolerance by the f-box protein MAX2 in arabidopsis. Plant Physiol. 164 (1), 424–439. doi: 10.1104/pp.113.226837 24198318 PMC3875819

[B7] Cannon S. B. Mitra A. Baumgarten A. Young N. D. May G. (2004). The roles of segmental and tandem gene duplication in the evolution of large gene families in arabidopsis thaliana. BMC Plant Biol. 4, 10. doi: 10.1186/1471-2229-4-10 15171794 PMC446195

[B8] Carafoli E. Krebs J. (2016). Why calcium? how calcium became the best communicator. J. Biol. Chem. 291 (40), 20849–20857. doi: 10.1074/jbc.R116.735894 27462077 PMC5076498

[B9] Chen X. Chen Z. Zhao H. Zhao Y. Cheng B. Xiang Y. (2014). Genome-wide analysis of soybean HD-zip gene family and expression profiling under salinity and drought treatments. PloS One 9 (2), e87156. doi: 10.1371/journal.pone.0087156 24498296 PMC3911943

[B10] Chin D. Means A. R. (2000). Calmodulin: a prototypical calcium sensor. Trends Cell Biol. 10 (8), 322–328. doi: 10.1016/S0962-8924(00)01800-6 10884684

[B11] DeFalco T. A. Bender K. W. Snedden W. A. (2009). Breaking the code: Ca^2+^ sensors in plant signalling. Biochem. J. 425 (1), 27–40. doi: 10.1042/BJ20091147 20001960

[B12] Dröge-Laser W. Snoek B. L. Snel B. Weiste C. (2018). The arabidopsis bZIP transcription factor family-an update. Curr. Opin. Plant Biol. 45 (Pt A), 36–49. doi: 10.1016/j.pbi.2018.05.001 29860175

[B13] Edel K. H. Marchadier E. Brownlee C. Kudla J. Hetherington A. M. (2017). The evolution of calcium-based signalling in plants. Curr. biology: CB 27 (13), R667–R679. doi: 10.1016/j.cub.2017.05.020 28697370

[B14] Fan T. Lv T. Xie C. Zhou Y. Tian C. (2021). Genome-wide analysis of the *IQM* gene family in rice (*Oryza sativa* l.). Plants (Basel Switzerland) 10 (9), 1949. doi: 10.3390/plants10091949 34579481 PMC8469326

[B15] Feng L. Chen Z. Ma H. Chen X. Li Y. Wang Y. . (2014). The IQD gene family in soybean: structure, phylogeny, evolution and expression. PloS One 9 (10), e110896. doi: 10.1371/journal.pone.0110896 25343341 PMC4208818

[B16] Flagel L. E. Wendel J. F. (2009). Gene duplication and evolutionary novelty in plants. New Phytol. 183 (3), 557–564. doi: 10.1111/j.1469-8137.2009.02923.x 19555435

[B17] Gong L. Chen J. Zhou Y. Huang X. Tian C. E. (2016). Disruption of IQM5 delays flowering possibly through modulating the juvenile-to-adult transition. Acta Physiologiae Plantarum 39, 21. doi: 10.1007/s11738-016-2314-4

[B18] Hurst L. D. (2002). The Ka/Ks ratio: diagnosing the form of sequence evolution. Trends genetics: TIG 18 (9), 486. doi: 10.1016/s0168-9525(02)02722-1 12175810

[B19] Hyun T. K. Eom S. H. Han X. Kim J. S. (2014). Evolution and expression analysis of the soybean glutamate decarboxylase gene family. J. Biosci. 39 (5), 899–907. doi: 10.1007/s12038-014-9484-2 25431418

[B20] Jakoby M. Weisshaar B. Dröge-Laser W. Vicente-Carbajosa J. Tiedemann J. Kroj T. . (2002). bZIP transcription factors in arabidopsis. Trends Plant Sci. 7 (3), 106–111. doi: 10.1016/S1360-1385(01)02223-3 11906833

[B21] Li Y. Chen Q. Nan H. Li X. Lu S. Zhao X. . (2017). Overexpression of GmFDL19 enhances tolerance to drought and salt stresses in soybean. PloS One 12 (6), e0179554. doi: 10.1371/journal.pone.0179554 28640834 PMC5480881

[B22] Li K. Ma B. Shen J. Zhao S. Ma X. Wang Z. . (2021). The evolution of the expansin gene family in brassica species. Plant Physiol. biochemistry: PPB 167, 630–638. doi: 10.1016/j.plaphy.2021.08.033 34479031

[B23] Lv T. Li X. Fan T. Luo H. Xie C. Zhou Y. . (2019). The calmodulin-binding protein IQM1 interacts with CATALASE2 to affect pathogen defense. Plant Physiol. 181 (3), 1314–1327. doi: 10.1104/pp.19.01060 31548265 PMC6836832

[B24] Lynch M. Conery J. S. (2000). The evolutionary fate and consequences of duplicate genes. Sci. (New York N.Y.) 290 (5494), 1151–1155. doi: 10.1126/science.290.5494.1151 11073452

[B25] Ma H. Feng L. Chen Z. Chen X. Zhao H. Xiang Y. (2014). Genome-wide identification and expression analysis of the IQD gene family in populus trichocarpa. Plant science: an Int. J. Exp. Plant Biol. 229, 96–110. doi: 10.1016/j.plantsci.2014.08.017 25443837

[B26] Mei C. Liu Y. Dong X. Song Q. Wang H. Shi H. . (2021). Genome-wide identification and characterization of the potato *IQD* family during development and stress. Front. Genet. 12, 693936. doi: 10.3389/fgene.2021.693936 34386041 PMC8354571

[B27] Mengarelli D. A. Zanor M. I. (2021). Genome-wide characterization and analysis of the CCT motif family genes in soybean (Glycine max). Planta 253 (1), 15. doi: 10.1007/s00425-020-03537-5 33392793

[B28] Meng X. Wang C. Rahman S. U. Wang Y. Wang A. Tao S. (2015). Genome-wide identification and evolution of HECT genes in soybean. Int. J. Mol. Sci. 16 (4), 8517–8535. doi: 10.3390/ijms16048517 25894222 PMC4425094

[B29] Nekrutenko A. Makova K. D. Li W. H. (2002). The K(A)/K(S) ratio test for assessing the protein-coding potential of genomic regions: an empirical and simulation study. Genome Res. 12 (1), 198–202. doi: 10.1101/gr.200901 11779845 PMC155263

[B30] Panchy N. Lehti-Shiu M. Shiu S. H. (2016). Evolution of gene duplication in plants. Plant Physiol. 171 (4), 2294–2316. doi: 10.1104/pp.16.00523 27288366 PMC4972278

[B31] Ranty B. Aldon D. Cotelle V. Galaud J. P. Thuleau P. Mazars C. (2016). Calcium sensors as key hubs in plant responses to biotic and abiotic stresses. Front. Plant Sci. 7, 327. doi: 10.3389/fpls.2016.00327 27014336 PMC4792864

[B32] Reddy A. S. Ali G. S. Celesnik H. Day I. S. (2011). Coping with stresses: roles of calcium- and calcium/calmodulin-regulated gene expression. Plant Cell 23 (6), 2010–2032. doi: 10.1105/tpc.111.084988 21642548 PMC3159525

[B33] Reddy A. S. Day I. S. (2001). Analysis of the myosins encoded in the recently completed arabidopsis thaliana genome sequence. Genome Biol. 2 (7), RESEARCH0024. doi: 10.1186/gb-2001-2-7-research0024 11516337 PMC55321

[B34] Rhoads A. R. Friedberg F. (1997). Sequence motifs for calmodulin recognition. FASEB journal: Off. Publ. Fed. Am. Societies Exp. Biol. 11 (5), 331–340. doi: 10.1096/fasebj.11.5.9141499 9141499

[B35] Saijo Y. Loo E. P. (2020). Plant immunity in signal integration between biotic and abiotic stress responses. New Phytol. 225 (1), 87–104. doi: 10.1111/nph.15989 31209880

[B36] Sankoff D. (2001). Gene and genome duplication. Curr. Opin. Genet. Dev. 11 (6), 681–684. doi: 10.1016/S0959-437X(00)00253-7 11682313

[B37] Schmutz J. Cannon S. B. Schlueter J. Ma J. Mitros T. Nelson W. . (2010). Genome sequence of the palaeopolyploid soybean. Nature 463 (7278), 178–183. doi: 10.1038/nature08670 20075913

[B38] Talke I. N. Blaudez D. Maathuis F. J. Sanders D. (2003). CNGCs: prime targets of plant cyclic nucleotide signalling? Trends Plant Sci. 8 (6), 286–293. doi: 10.1016/S1360-1385(03)00099-2 12818663

[B39] Truman W. Sreekanta S. Lu Y. Bethke G. Tsuda K. Katagiri F. . (2013). The CALMODULIN-BINDING PROTEIN 60 family includes both negative and positive regulators of plant immunity. Plant Physiol. 163 (4), 1741–1751. doi: 10.1104/pp.113.227108 24134885 PMC3850189

[B40] Wang M. Chen B. Zhou W. Xie L. Wang L. Zhang Y. . (2021). Genome-wide identification and expression analysis of the AT-hook motif nuclear localized gene family in soybean. BMC Genomics 22 (1), 361. doi: 10.1186/s12864-021-07687-y 34006214 PMC8132359

[B41] Wang L. Guo K. Li Y. Tu Y. Hu H. Wang B. . (2010). Expression profiling and integrative analysis of the CESA/CSL superfamily in rice. BMC Plant Biol. 10, 282. doi: 10.1186/1471-2229-10-282 21167079 PMC3022907

[B42] Wang X. Wu M. H. Xiao D. Huang R. L. Zhan J. Wang A. Q. . (2021). Genome-wide identification and evolutionary analysis of RLKs involved in the response to aluminium stress in peanut. BMC Plant Biol. 21 (1), 281. doi: 10.1186/s12870-021-03031-4 34154532 PMC8215822

[B43] Williams R. J. (1992). Calcium and calmodulin. Cell calcium 13 (6-7), 355–362. doi: 10.1016/0143-4160(92)90049-X 1505001

[B44] Wu M. Li Y. Chen D. Liu H. Zhu D. Xiang Y. (2016). Genome-wide identification and expression analysis of the IQD gene family in moso bamboo (Phyllostachys edulis). Sci. Rep. 6, 24520. doi: 10.1038/srep24520 27094318 PMC4837358

[B45] Yang T. Poovaiah B. W. (2003). Calcium/calmodulin-mediated signal network in plants. Trends Plant Sci. 8 (10), 505–512. doi: 10.1016/j.tplants.2003.09.004 14557048

[B46] Zhang X. Feng Y. Cheng H. Tian D. Yang S. Chen J. Q. (2011). Relative evolutionary rates of NBS-encoding genes revealed by soybean segmental duplication. Mol. Genet. genomics: MGG 285 (1), 79–90. doi: 10.1007/s00438-010-0587-7 21080199

[B47] Zhang M. Liu Y. Shi H. Guo M. Chai M. He Q. . (2018). Evolutionary and expression analyses of soybean basic leucine zipper transcription factor family. BMC Genomics 19 (1), 159. doi: 10.1186/s12864-018-4511-6 29471787 PMC5824455

[B48] Zhang Z. Zhao Y. Feng X. Luo Z. Kong S. Zhang C. . (2019). Genomic, molecular evolution, and expression analysis of NOX genes in soybean (Glycine max). Genomics 111 (4), 619–628. doi: 10.1016/j.ygeno.2018.03.018 29621573

[B49] Zhang H. Zhu J. Gong Z. Zhu J. K. (2022). Abiotic stress responses in plants. Nat. Rev. Genet. 23 (2), 104–119. doi: 10.1038/s41576-021-00413-0 34561623

[B50] Zhao W. Cheng Y. H. Zhang C. Shen X. J. You Q. B. Guo W. . (2017). Genome-wide identification and characterization of the GmSnRK2 family in soybean. Int. J. Mol. Sci. 18 (9), 1834. doi: 10.3390/ijms18091834 28832544 PMC5618483

[B51] Zhao K. Chen S. Yao W. Cheng Z. Zhou B. Jiang T. (2021). Genome-wide analysis and expression profile of the bZIP gene family in poplar. BMC Plant Biol. 21 (1), 122. doi: 10.1186/s12870-021-02879-w 33648455 PMC7919096

[B52] Zhou Y. Chen Y. Yamamoto K. T. Duan J. Tian C. E. (2010). Sequence and expression analysis of the arabidopsis IQM family. Acta Physiologiae Plantarum 32 (1), 191–198. doi: 10.1007/s11738-009-0398-9

[B53] Zhou Y. Duan J. Fujibe T. Yamamoto K. T. Tian C. E. (2012). AtIQM1, a novel calmodulin-binding protein, is involved in stomatal movement in arabidopsis. Plant Mol. Biol. 79 (4-5), 333–346. doi: 10.1007/s11103-012-9915-0 22572939

[B54] Zhou Z. Lakhssassi N. Knizia D. Cullen M. A. El Baz A. Embaby M. G. . (2021). Genome-wide identification and analysis of soybean acyl-ACP thioesterase gene family reveals the role of GmFAT to improve fatty acid composition in soybean seed. Theor. Appl. Genet. 134 (11), 3611–3623. doi: 10.1007/s00122-021-03917-9 34319424

[B55] Zhou Y. Wu J. Xiao W. Chen W. Chen Q. Fan T. . (2018). *Arabidopsis* IQM4, a novel calmodulin-binding protein, is involved with seed dormancy and germination in arabidopsis. Front. Plant Sci. 9, 721. doi: 10.3389/fpls.2018.00721 29951071 PMC6008652

[B56] Zhu T. Liu Y. Ma L. Wang X. Zhang D. Han Y. . (2020). Genome-wide identification, phylogeny and expression analysis of the SPL gene family in wheat. BMC Plant Biol. 20 (1), 420. doi: 10.1186/s12870-020-02576-0 32912142 PMC7488452

