# Supplementary material for: Genome-wide identification and analysis of the IQM gene family in soybean
Source: Front Plant Sci. 2023 Jan 6;13:1093589. doi: 10.3389/fpls.2022.1093589 (PMC9853202; doi:10.3389/fpls.2022.1093589)
Supplement: Supplementary file 1 [file DataSheet_1.docx]

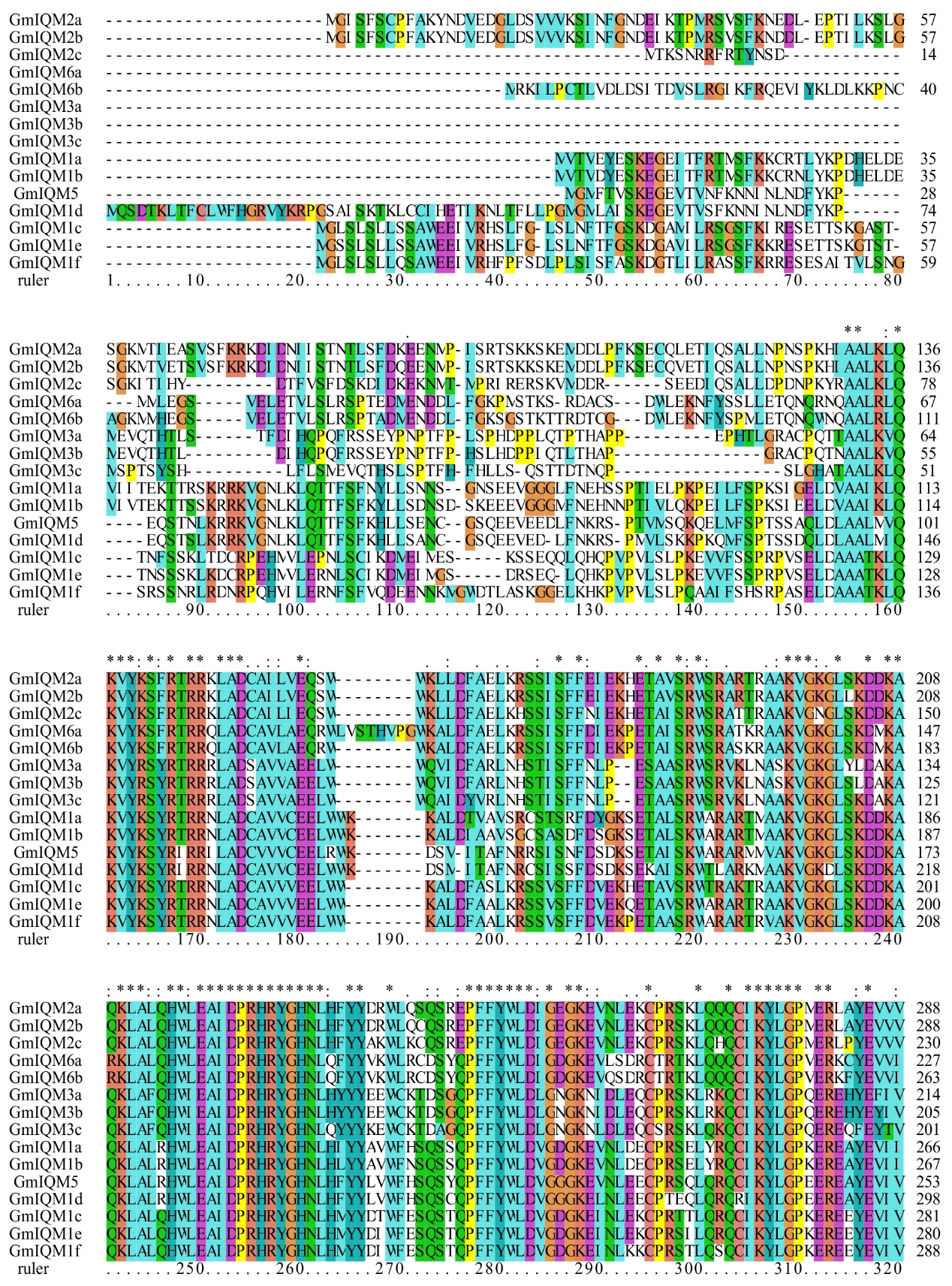
**FIGURE S1|** The amino acid sequence alignments of IQM protein in soybean. Asterisks indicate conserved amino acids in the sequence


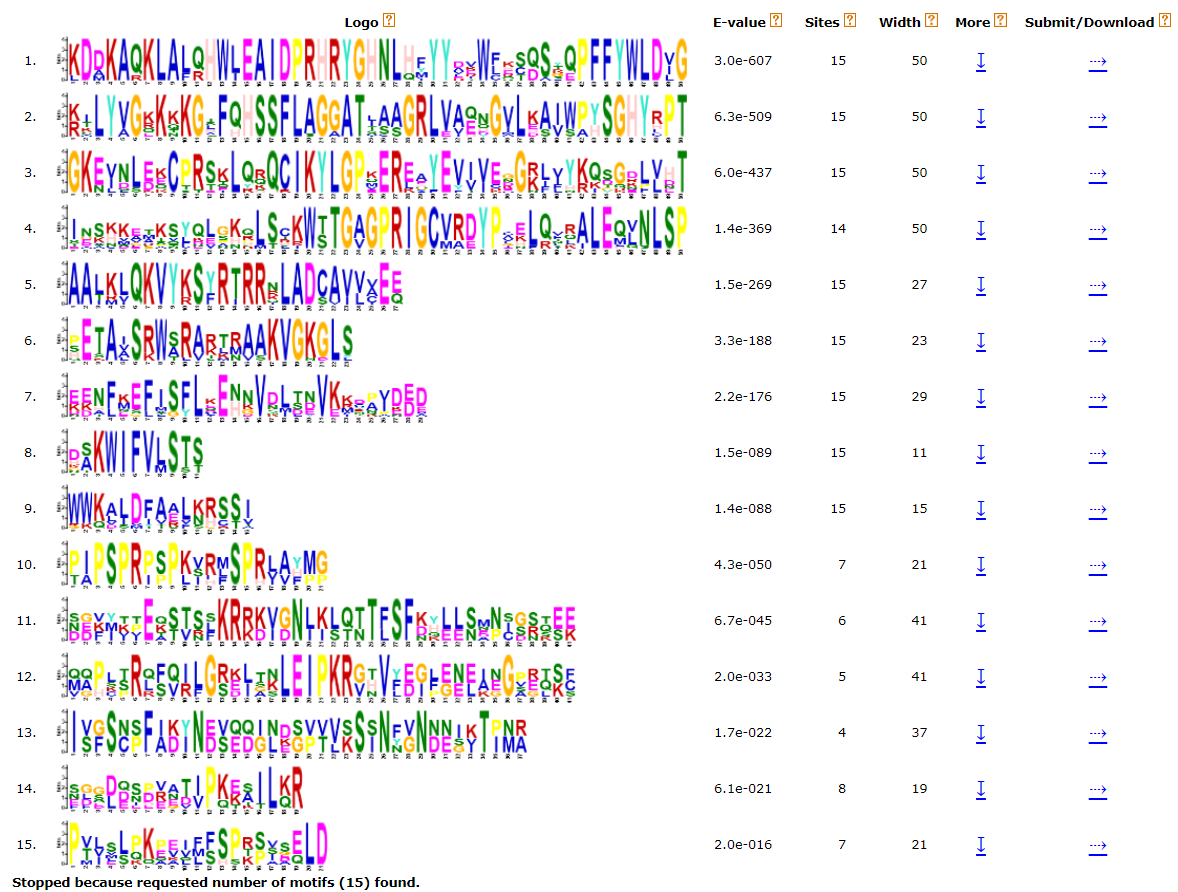


**FIGURE S2|** The conserved amino acid sequences of each motif in soybean IQM proteins.

**TABLE S1|** IQ motif amino acid sequence of 15 soybean IQM genes.

| **Group** | **Name** | **Gene ID** | **IQ motif** |
| --- | --- | --- | --- |
| I | GmIQM1a | Glyma.07G008300 | LQKVYKSYRTRRNLA |
| I | GmIQM1b | Glyma.08G191300 | LQKVYKSYRTRRNLA |
| I | GmIQM1c | Glyma.09G065000 | LQKVYKSYRTRRNLA |
| I | GmIQM1d | Glyma.15G025300 | IQKVYKSYRIRRNLA |
| I | GmIQM1e | Glyma.15G171000 | LQKVYKSYRTRRNLA |
| I | GmIQM1f | Glyma.17G053600 | LQKVYKSYRTRRNLA |
| II | GmIQM2a | Glyma.05G156600 | LQKVYKSFRTRRKLA |
| II | GmIQM2b | Glyma.08G114700 | LQKVYKSFRTRRKLA |
| II | GmIQM2c | Glyma.18G000400 | LQKVYKSFRTRRKLA |
| III | GmIQM3a | Glyma.10G067900 | VQKVYRSYRTRRRLA |
| III | GmIQM3b | Glyma.11G212100 | VQKVYRSYRTRRRLA |
| III | GmIQM3c | Glyma.19G193300 | LQKVYRSYRTRRRLA |
| I | GmIQM5 | Glyma.13G348500 | VQKVYKSYRIRRILA |
| II | GmIQM6a | Glyma.02G168100 | LQKVYKSFRTRRQLA |
| II | GmIQM6b | Glyma.09G103600 | LQKVYKSFRTRRQLA |

**TABLE S2|** List of the qRT-PCR primers in this study.

| **Gene ID** | Primer name | **Sequences 5’→3’** |
| --- | --- | --- |
| Glyma.07G008300 | GmIQM1a-qRT-F | ACTTTCAAATAACTCTGGTAAT |
|  | GmIQM1a-qRT-R | CGTGACGTGGAACACCGA |
| Glyma.08G191300 | GmIQM1b-qRT-F | TGAGATAGACTTCCACCCGT |
|  | GmIQM1b-qRT-R | ACCATTTGCACACCTCTCTT |
| Glyma.09G065000 | GmIQM1c-qRT-F | GTGCCTCTACCACCAACTTT |
|  | GmIQM1c-qRT-R | GCTGCAATTGTTGTTCACTGC |
| Glyma.15G025300 | GmIQM1d-qRT-F | ATGCAATCGGACACTAAACTC |
|  | GmIQM1d-qRT-R | CTTCAGATTTCCCACCTTCC |
| Glyma.15G171000 | GmIQM1e-qRT-F | TCGCTCCAGTTCTTGACGT |
|  | GmIQM1e-qRT-R | GATCCTTGGGCTGGGCAA |
| Glyma.17G053600 | GmIQM1f-qRT-F | CCTGTGCTTTCTCTGCCTCA |
|  | GmIQM1f-qRT-R | AACCTTTGCCGACCTTAGCT |
| Glyma.05G156600 | GmIQM2a-qRT-F | ATTAATAGCAAAGGATCCCATT |
|  | GmIQM2a-qRT-R | TAAGATATGAAATGATAGTGTTC |
| Glyma.08G114700 | GmIQM2b-qRT-F | ATCACACACAGGTAGTCAAGA |
|  | GmIQM2b-qRT-R | GTCCCCACATAAACTTGCCA |
| Glyma.18G000400 | GmIQM2c-qRT-F | AGTTGGACCTCGTATTGGCT |
|  | GmIQM2c-qRT-R | GCTCAAGTCCTCGAAGCAGA |
| Glyma.10G067900 | GmIQM3a-qRT-F | ACCCTCTCTTTCTCTGTGTGA |
|  | GmIQM3a-qRT-R | CGAAGGTGGAGAGAGTGTGA |
| Glyma.11G212100 | GmIQM3b-qRT-F | GGAAGCTGACAACACGACAT |
|  | GmIQM3b-qRT-R | TGCCATTGGTCGTAGGTGG |
| Glyma.19G193300 | GmIQM3c-qRT-F | CCGACACCAACCAACCTTCT |
|  | GmIQM3c-qRT-R | GCAGCGTTCAATTTAACCCGA |
| Glyma.13G348500 | GmIQM5-qRT-F | TTTCGGTCAACTTGTCGTCG |
|  | GmIQM5-qRT-R | GCAGGAATGGATGACAGCAC |
| Glyma.02G168100 | GmIQM6a-qRT-F | CCTCTCTCTCTGCACCAACA |
|  | GmIQM6a-qRT-R | GTGCAGCTTGGTTCCTTTGA |
| Glyma.09G103600 | GmIQM6b-qRT-F | ATGAGAAAAATCCTACCTTGCAC |
|  | GmIQM6b-qRT-R | GCACAGTTTGGTTTCTTTAAGTC |
| Glyma.05G207500 | GmTublin-F | GAGAAGAGTATCCGGATAGG |
|  | GmTublin-R | GTTTCCGAACACTCAAGCTC |
